# Geographic and ethnic differences in childhood leukaemia and lymphoma survival: comparisons of Philippine residents, Asian Americans and Caucasians in the United States

**DOI:** 10.1038/sj.bjc.6605703

**Published:** 2010-05-18

**Authors:** M T Redaniel, A Laudico, M R Mirasol-Lumague, A P Alcasabas, D Pulte, H Brenner

**Affiliations:** 1 Division of Clinical Epidemiology and Aging Research, German Cancer Research Center, Bergheimer Str 20, Heidelberg D-69115, Germany; 2 Manila Cancer Registry, Philippine Cancer Society, 310 San Rafael St, San Miguel, Manila 1005, Philippines; 3 Department of Surgery, Philippine General Hospital, University of the Philippines-Manila, Taft Avenue, Manila 1000, Philippines; 4 Department of Health-Rizal Cancer Registry, Rizal Medical Center, Pasig Boulevard, Pasig City 1600, Philippines; 5 Department of Pediatrics, Philippine General Hospital, University of the Philippines-Manila, Taft Avenue, Manila 1000, Philippines; 6 Department of Paediatrics, University Children's Medical Institute, National University Hospital, 5 Lower Kent Ridge Road, Singapore 119074, Singapore; 7 Division of Hematology/Oncology, Department of Medicine, University of Medicine and Dentistry of New Jersey, Newark, NJ 07101, USA

**Keywords:** childhood, leukaemia, lymphoma, survival

## Abstract

**Background:**

Childhood cancer survival estimates from developing nations are rare.

**Methods:**

Using the US SEER and the Manila and Rizal Cancer Registry databases in the Philippines, 5-year survival for childhood leukaemia and lymphoma in 2001–2005 among Asian Americans were compared with both Filipinos and Caucasians in the United States. Estimates for patients in the United States in earlier time periods were compared with that of Philippine residents to estimate delay in achievements of comparable levels of survival.

**Results:**

Childhood leukaemia and lymphoma relative survival was much lower in Filipinos living in the Philippines (32.9 and 47.7%) than in Asian Americans (80.1 and 90.5%) and Caucasians (81.9 and 87%). Achievement of comparable survival rates of Philippine residents lagged behind by 20 to >30 years compared with patients in the United States.

**Conclusions:**

The large differences in survival estimates of US populations and Philippine residents highlight the deficiencies of paediatric cancer care delivery in the Philippines. The long survival lag underlines the need for major improvements in access to diagnostic and treatment facilities.

Childhood cancer survival rates vary in different countries and ethnicities (Stiller *et al*, 2000; Bhatia *et al*, 2002; Gatta *et al*, 2002, 2003; Kadan-Lottick *et al*, 2003; Aplenc *et al*, 2006; Brenner *et al*, 2007; Linabery and Ross, 2008). Nevertheless, international (Gatta *et al*, 2002, 2003; Brenner *et al*, 2007) and ethnic (Stiller *et al*, 2000; Bhatia *et al*, 2002; Kadan-Lottick *et al*, 2003; Aplenc *et al*, 2006; Linabery and Ross, 2008) comparisons are relatively few, particularly with low-income countries, mainly because of the absence of data.

Survival comparisons between countries and ethnic groups are important to assess the magnitude of survival discrepancies and to examine the possible sources of variability. By comparing survival estimates of developing countries with those from developed nations along time periods, delay in achieving comparable survival levels can be quantified.

In this study, we provide up-to-date estimates of childhood leukaemia and lymphoma survival for the Philippine resident population and Asian Americans, who have similar ethnicity, and Asian Americans and Caucasians from the United States, who have the same health-care system. To quantify delay in survival and determine lag time, 5-year estimates for various time periods between 1976–1980 and 2001–2005 were computed for children in the United States, and compared with the 2001–2005 survival estimates for Philippine residents.

## Materials and methods

US data were abstracted from the database of the Surveillance, Epidemiology and End Results (SEER) Program Limited-Use Data (1973–2005) (SEER, 2008), which routinely collects data on cancer patients from selected populations in the United States. For this analysis, the SEER 9 and 17 databases were used. From the SEER 17 database, patients 0–14 years of age, of either Asian-American or Caucasian origin, diagnosed with leukaemia or lymphoma based on the International Classification of Childhood Cancer (ICCC) definition (Steliarova-Foucher *et al*, 2005) between 1 January 1996 and 31 December 2005 were included. Death certificate only (DCO), *in situ* and autopsy cases were excluded. A total of 625 Asian-American and 6834 Caucasian children were included, comprising of 496 (79%) leukaemia and 129 (21%) lymphoma cases from the former, and 5344 (78%) leukaemia and 1490 (22%) lymphoma cases, from the latter group. In this study, Filipino Americans were combined with other Asian-American groups, because of their small numbers. From the SEER 9 database, patients 0–14 years of age, regardless of race, and diagnosed with ICCC classified leukaemia or lymphoma between 1 January 1976 and 31 December 2005 were included. DCO, *in situ* and autopsy cases were likewise excluded, leaving 6913 leukaemia and 2253 lymphoma cases for the analysis.

The databases used in the analyses of the Philippine resident population came from the Philippine Cancer Society–Manila Cancer Registry (PCS–MCR) and the Department of Health–Rizal Cancer Registry (DOH–RCR). These registries cover the National Capital Region (NCR), more commonly known as Metro Manila, which is the largest urban metropolis, and the political, social, educational and economic centre. This population is wealthier and has better access to health-care and cancer diagnostic and treatment facilities than the rest of the country (NSCB, 2006). The Philippine registries follow the cancer registration definitions and data collection guidelines set by the International Agency for Research on Cancer and the International Association of Cancer Registries. They are regarded as among the high-quality cancer registries from developing countries and have been regularly included in Cancer Incidence in Five Continents (Muir *et al*, 1987; Parkin *et al*, 1992, 1997, 2002; Curado *et al*, 2007).

From a list of patients diagnosed between 1 January 1996 and 31 December 2005, subjects were selected using the same inclusion and exclusion criteria as for the SEER 17 database. Survival status was assessed from death certificate notifications mentioning cancer as the cause of death, which were collected from the Local Civil Registry Offices. For those not identified as dead, active follow-up by personal visits to the patients or their families in the last known place of residence was used to confirm vital status.

From the 1500 childhood leukaemia and lymphoma cases registered in the PCS–MCR and DOH–RCR databases, 4 (<1%) were excluded because of invalid data, and 232 (16%) because of missing follow-up information. A total of 1264 (84%) patients were left in the analysis, for whom anonymised data sets were prepared. Of the cases, 86% (1081) had leukaemia while the remaining 14% (183) had lymphoma.

The project proposal was approved by the ethics review board of the National Institutes of Health of the University of the Philippines Manila. The information obtained strictly conformed to the code of conduct stipulated by the Guidelines on Confidentiality for Population-Based Cancer Registries (International Association of Cancer Registries & International Agency for Research on Cancer, 2004).

### Data analysis

In this study, period analysis of survival analysis was used (Brenner and Gefeller, 1996). With this approach, only the survival experience of patients for the 2001–2005 period was included. It has been shown that period analysis provides more up-to-date estimates of survival that closely predict survival later observed for patients diagnosed in the relevant period (Brenner *et al*, 2002; Brenner and Hakulinen, 2002a, 2002b; Tälback *et al*, 2004; Ellison, 2006; Steliarova-Foucher *et al*, 2007). Analyses were carried out by types of leukaemia ((Ia) ALL, (Ib) acute myeloid (AML), and other types, including (Ic) chronic myeloproliferative diseases, (Id) myelodysplastic syndrome and other myeloproliferative diseases and (Ie) unspecified and other leukaemias) and lymphomas ((IIa) Hodgkins (HL), (IIb) non-Hodgkin (NHL), and other types, including (IIc) Burkitt, (IId) miscellaneous lymphoreticular neoplasms and (IIe) unspecified lymphomas) (Steliarova-Foucher *et al*, 2005), by sex and by age (<1, 1–4, 5–9 and 10–14). Owing to small sample sizes for the other subtypes, only the survival estimates for ALL, AML and NHL were separately computed.

Estimates of relative survival (calculated as the ratio of observed and expected survival) were derived using the so-called Ederer II method (Ederer and Heise, 1959) and life tables for the year 2000. Owing to the absence of life tables for other races, the life table for whites from the US National Center for Health Statistics (Arias, 2002) was used for both SEER populations. The life table for the Philippine resident population was derived from the projected population estimate and the actual mortality data for this area, obtained from the Philippine National Statistics Office. To compare overall survival estimates between the different cancer populations, age was adjusted using the distribution of the Filipinos patients. The age groups used were <1, 1–4, 5–9 and 10–14.

To test for differences in survival between the ethnic groups, a novel modelling approach for period analysis (Brenner and Hakulinen, 2006) was used. First, age-specific numbers of patients at risk and of deaths by year of follow-up were calculated separately for each population group. Then, Poisson regression models were fitted, in which the numbers of deaths were modelled as a function of the group (Philippine residents or Asian Americans or US Caucasians) and year of follow-up (1, 2, 3, 4, 5 – entered as a categorical variable), using the logarithm of the person-years at risk as offset, and accounting for late entries and withdrawals as half persons, as described in detail elsewhere (Brenner and Hakulinen, 2006). In models for all types of leukaemia and lymphoma (both sexes and separately for men and women), and for ALL, AML and NHL, we also included age groups. This approach allowed testing for significance of differences in survival, after accounting for differences in distribution of subgroups, based on *P*-values for the population parameter estimate. A significance level of =0.05 (two-sided testing) was used.

Using the SEER 9 database, period survival estimates for the years 1976–1980, 1981–1985, 1986–1990, 1991–1995, 1996–2000 and 2001–2005 were derived for children in the United States, using the same algorithms used for the Philippine residents, Asian Americans and US Caucasians. These survival estimates were then compared with those for the Philippine population in 2001–2005, using only the data from the SEER 9 registries, as these have information dating back to the early 1970s. The other SEER registries were only included in the programme in the 1990s and 2001 (National Cancer Institute, September 2005).

## Results

In all three populations and in both sexes, a majority of leukaemia cases were diagnosed as ALL (Table 1), whereas NHL was the commonest lymphoma subtype. However, the proportion of leukaemia patients with ALL was lower, and of patients with undifferentiated leukaemias and AML was higher in the Philippine resident population than in the United States (*P*<0.0001). Among lymphoma patients, the proportion of children with HL was lower, and the proportion of children with other types of lymphoma was larger in Philippine residents (*P*<0.0001). More men were diagnosed than women in all groups for both types of malignancies. For leukaemias, the differences in age distribution between the three populations were not large (Table 2). For lymphomas, while most cases were older than 5 years in all groups, Philippine residents were relatively younger than the SEER populations, with a larger proportion (29.5%) of cases aged 4 years and below.

Age-adjusted, and type-, age- and sex-specific estimates of 5-year absolute and relative survival for the 2001–2005 period are shown in Table 3. Much lower leukaemia relative survival was observed in Filipinos living in the Philippines (32.9%) than in Asian Americans (80.1%) and Caucasians (81.9%). Children with ALL have better survival than those with AML for all populations, but this advantage is small among Philippine residents. Survival estimates for men are slightly higher than for women in all three groups. Stratification by age reveals that the survival disadvantage of Philippine residents is particularly large for age group 10–14 years, where 5-year relative survival was as low as 17.6%. For lymphoma, and NHL in particular, relative survival estimates for Philippine residents were likewise much lower (47.7 and 49.7%, for lymphoma and NHL, respectively) than the US populations (90.5 and 84.8% for Asian Americans, and 87.0 and 81.0% for Caucasians).

Trends in 5-year survival for childhood leukaemia and lymphoma cases in the United States between 1976–1980 and 2001–2005 is shown in Table 4. For all groups, rates strongly increased over time, with improvements between the 1976–1980 and 2001–2005 periods ranging from 25 to 40%. When compared with Philippine residents, survival estimates for patients from the Philippines in 2001–2005 were even lower than those for the US populations in 1976–1980, with the exception of AML, for which survival in the Philippines in 2001–2005 was similar to that for American children in 1981–1990.

## Discussion

In this comparative analysis of childhood leukaemia and lymphoma in 2001–2005, a strong survival disadvantage was observed for Philippine residents as compared with US children, while survival among Asian-American and Caucasian children was similar. Although estimates for Philippine children were expected to be lower than US patients, a lag in achievement of comparable survival rates of 20 to >30 years is disappointing. These survival rates highlight the deficiencies of paediatric cancer care in the Philippines, including unavailability of and inadequate access to diagnostic and treatment facilities, financial difficulties and lack of awareness.

In the Philippines, while state-of-the-art diagnostic and treatment modalities, such as immunophenotyping, cytogenetics, linear accelerator, MRI and PET–CT scan are present, these are inaccessible to most of the population. Services are available in paediatric oncology sections of large tertiary hospitals, which are few, mainly situated in major cities, and sometimes understaffed. Although there are adequate numbers of paediatric haematologists or oncologists in the country, there is no certified paediatric cancer unit (Ribeiro *et al*, 2008). A majority of the institutes and hospitals do not have full time paediatric haematologists–oncologists and specialised paediatric oncology nurses (Corrigan and Feig, 2004). Outside these units, diagnostic testing was described as nonexistent to limited, with a long wait for results at best, and availability of medications and blood products were limited or irregular, making supportive care for cancer patients poor (Ribeiro *et al*, 2008).

The expensive nature of paediatric cancer care further complicates the problem of inadequate health services and staff. The Philippines has a 2008 Gross National Income per capita of USD 3900 as compared with USD 46 970 for the United States (World Bank, 2009). Although a national health-care insurance, PhilHealth, was introduced in 1997, its coverage is limited and most patients are not enrolled. Moreover, most patients cannot start treatment, and for those who do, treatment abandonment is common. In contrast, almost all children with cancer in the United States are treated in highly specialised paediatric oncology centres, which are a part of a national children oncology group. Furthermore, a special insurance programme covering children with serious illnesses, including cancer, is available (Centers for Medicare and Medicaid Services, 2009), resulting in a lower frequency of treatment discontinuation.

Lack of awareness is an important issue in the Philippines, especially on health problems relating to children, which leads to delays in seeking medical help and treatment (Usmani, 2001). Owing to the nonspecificity of signs and symptoms of early childhood leukaemia and lymphoma, most patients from developing countries are diagnosed at a later stage. For example, data from the Philippine General Hospital show that 55.3% of newly diagnosed paediatric charity ALL cases diagnosed in 2003–2007 were high risk by US National Cancer Institute criteria, with more than half having an initial white blood cell count >50 000 (Lubaton *et al*, 2009). Furthermore, 71% of newly diagnosed solid tumour patients in 2008 presented at stages III and IV. Similarly, inadequate education and poverty compel some parents to turn to traditional and alternative medicines for treatment (Usmani, 2001; Arora *et al*, 2007).

Similar survival estimates were observed between Asian Americans and Caucasians, consistent with previous findings (Kadan-Lottick *et al*, 2003). This could be attributed to the high level of health-care access among Asian Americans, the majority of whom being either native born (31%) or naturalised citizens (34%) (Reeves and Bennett, 2004). Moreover, Asian Americans have higher earnings than the average American, with a large proportion having attained a bachelor's degree or more (44%), and more likely to be in management, professional or related occupations (45%) (Reeves and Bennett, 2004).

Taken together, our results underline the overwhelming importance of access to effective treatment as accounting for the observed survival differences between the populations studied. Even though some ethnic variability has been observed, under trial conditions with identical treatment and access (Aplenc *et al*, 2006), this variation was small compared with the huge variation according to the health-care system in our study.

Some limitations of our study are relevant because not all factors that could affect survival could be considered. Information not obtainable include factors related to cancer services, such as training and skills of health-care professionals, accuracy of diagnostic tests, chemotherapy protocols used, and disease characteristics such as cancer stage, and comorbidities. Furthermore, in spite of exhaustive measures to locate Philippine resident for survival information, including personal visits to the homes, follow-up was in complete for 25% of patients, attributable to the high migration and mobility in the study area. Nevertheless, it can be assumed that patients lost to follow-up have not received or have discontinued treatment, as follow-up information was not found or incomplete in hospitals within the NCR. The availability of cancer treatment is limited in the surrounding regions and it is unlikely that patients lost to follow-up have higher survival than those who were not.

In spite of these limitations, our results highlight the very large survival disadvantage of children in the Philippines. Efforts such as more twinning programmes between hospitals and specialised centres in developed and developing countries should be considered, as well as increased government spending on health, which have been shown to be effective in enhancing care of childhood leukaemia in developing countries (Pui and Ribeiro, 2003; Howard *et al*, 2004).

## Figures and Tables

**Table 1:** Tumour characteristics of childhood leukaemia and lymphoma patients among the Philippine resident population and Asian Americans and Caucasians from US SEER, 1996–2005

		**Philippine resident population**		**Asian American**		**Caucasians**		
**Sex**	**Type of malignancy**	**Freq**	**%**	**Freq**	**%**	**Freq**	**%**	***P*-value**
Both sexes	*Leukaemia*	1081		496		5344		<0.0001
	ALL	699	64.7	376	75.8	4350	81.4	
	AML	252	23.3	93	18.8	789	14.8	
	Other types	130	12.0	27	5.4	205	3.8	
	*Lymphoma*	183		129		1490		<0.0001
	HL	21	11.5	34	26.4	607	40.7	
	NHL	85	46.5	65	50.4	607	40.7	
	Other types	77	42.1	30	23.3	276	18.5	
								
Males	*Leukaemia*	639		285		2963		<0.0001
	ALL	429	67.1	220	77.2	2417	81.6	
	AML	135	21.1	51	17.9	426	14.4	
	Other types	75	11.7	14	4.9	120	4.1	
	*Lymphoma*	120		86		963		<0.0001
	HL	15	12.5	23	26.7	353	36.7	
	NHL	59	49.2	40	46.5	385	40.0	
	Other types	46	38.3	23	26.7	225	23.4	
								
Females	*Leukaemia*	442		211		2381		<0.0001
	ALL	270	61.1	156	73.9	1933	81.2	
	AML	117	26.5	42	19.9	363	15.3	
	Other types	55	12.4	13	6.2	85	3.6	
	*Lymphoma*	63		43		527		<0.0001
	HL	6	9.5	11	25.6	254	48.2	
	NHL	26	41.3	25	58.1	222	42.1	
	Other types	31	49.2	7	16.3	51	9.7	

Abbreviations: ALL=acute lymphoblastic leukaemia; AML=acute myeloblastic leukaemia; HL=Hodgkin lymphoma; NHL=non-Hodgkin lymphoma; SEER=surveillance, epidemiology and end results.

**Table 2:** Childhood leukaemia and lymphoma patients among the Philippine resident population and Asian Americans and Caucasians from US SEER, 1996–2005, by age group

	**Philippine resident population**		**Asian American**		**Caucasians**		
**Age group**	**Freq**	**%**	**Freq**	**%**	**Freq**	**%**	***P*-value**
*Leukaemia*	1081		496		5344		0.009
<1	67	6.2	34	6.9	319	6.0	
1–4	457	42.3	249	50.2	2527	47.3	
5–9	349	32.3	127	25.6	1455	27.2	
10–14	208	19.2	86	17.3	1043	19.5	
							
*ALL*	699		376		4350		<0.001
<1	38	5.4	18	4.8	142	3.3	
1–4	315	45.1	206	54.8	2211	50.8	
5–9	234	33.5	96	25.5	1241	28.5	
10–14	112	16.0	56	14.9	756	17.4	
							
*Lymphoma*	183		129		1490		<0.0001
<1	2	1.1	2	1.6	10	0.7	
1–4	52	28.4	26	20.2	190	12.8	
5–9	52	28.4	32	24.8	449	30.1	
10–14	77	42.1	69	53.5	841	56.4	

Abbrevaitions: ALL=acute lymphoblastic leukaemia; SEER=surveillance, epidemiology and end results.

**Table 3:** Five-year survival (in %) of childhood leukaemia and lymphoma patients, Philippine resident population and Asian Americans and Caucasians from US SEER, 2001–2005

	**(1) Philippine resident population**		**Between (1) and (2)**		**(2) Asian American**		**Between (3) and (2)**		**(3) Caucasians**	
**Variable**	**%**	**s.e.**	**Diff**	***P*-value**	**%**	**s.e.**	**Diff**	***P*-value**	**%**	**s.e.**
*Absolute survival*										
Leukaemias										
Overall^a^	32.3	2.6	47.7	<0.001	80.0	2.6	1.7	0.39	81.7	0.7
By type^a^										
ALL	33.8	3.1	53.3	<0.001	87.2	2.4	−1.1	0.9993	86.1	0.7
AML	28.2	5.7	33.5	<0.001	61.8	7.5	−2.0	0.86	59.7	2.5
By sex^a^										
Male	35.6	3.5	46.3	<0.001	81.9	3.5	−0.6	0.83	81.2	1.0
Female	27.3	3.8	51.0	<0.001	78.3	3.8	3.8	0.12	82.1	1.1
By age group										
<1	29.0	10.3	31.5	0.02	60.5	11.9	−3.0	0.72	57.4	3.7
1–4	37.8	4.3	44.2	<0.001	82.0	3.6	6.5	0.08	88.5	0.9
5–9	34.7	4.5	45.7	<0.001	80.4	5.0	1.9	0.43	82.3	1.4
10–14	17.5	4.7	63.8	<0.001	81.3	5.6	−7.6	0.53	73.7	1.9
										
Lymphomas										
Both sexes^a^	47.1	6.1	43.2	<0.001	90.3	2.6	−3.5	0.99	86.9	1.4
NHL^a^	49.1	9.3	35.6	<0.01	84.7	4.9	−3.9	0.85	80.8	2.1
										
*Relative survival*										
Leukaemias										
Overall^a^	32.9	2.6	47.3	<0.001	80.1	2.6	1.7	0.38	81.9	0.7
By type^a^										
ALL	34.4	3.1	52.9	<0.001	87.3	2.4	−1.1	0.995	86.2	0.7
AML	28.5	5.7	33.3	<0.001	61.9	7.5	−2.0	0.86	59.8	2.5
By sex^a^										
Male	36.2	3.6	45.8	<0.001	82.0	3.5	−0.6	0.83	81.4	1.0
Female	27.6	3.8	50.8	<0.001	78.4	3.8	3.8	0.12	82.2	1.1
By age group										
<1	30.3	10.7	30.6	<0.001	60.9	12.0	−3.1	0.26	57.9	3.7
1–4	38.7	4.4	43.4	<0.001	82.1	3.6	6.5	0.43	88.6	0.9
5–9	34.8	4.5	45.6	<0.001	80.4	5.0	1.9	0.43	82.3	1.4
10–14	17.6	4.7	63.9	<0.001	81.5	5.6	−7.6	0.53	73.8	1.9
Lymphomas										
Both sexes^a^	47.7	6.2	42.7	<0.001	90.5	2.6	−3.5	0.98	87.0	1.4
NHL^a^	49.7	9.4	35.1	<0.01	84.8	4.9	−3.8	0.85	81.0	2.2

Abbreviations: ALL=acute lymphoblastic leukaemia; AML=acute myeloblastic leukaemia; NHL=non-Hodgkin lymphoma; SEER=surveillance, epidemiology and end results.

a Adjusted to the age distribution of the leukaemia/lymphoma of the patients from the Philippines.

**Table 4:** Five-year survival (in %) of childhood leukaemia and lymphoma patients, US SEER 9, 1976–2005

	**1976–1980**		**1981–1985**		**1986–1990**		**1991–1995**		**1996–2000**		**2001–2005**	
**Variable**	**%**	**s.e.**	**%**	**s.e.**	**%**	**s.e.**	**%**	**s.e.**	**%**	**s.e.**	**%**	**s.e.**
*Absolute survival*												
Leukaemias												
Overall^a^	52.7	2.7	60.7	1.6	66.5	1.4	72.3	1.3	79.1	1.2	82.9	1.1
By type^a^												
ALL	60.7	3.1	67.4	1.7	74.2	1.5	79.1	1.4	85.6	1.1	88.1	1.0
AML	20.7	4.8	29.4	4.4	35.8	4.4	40.5	3.8	48.9	3.7	60.7	3.7
By sex^a^												
Male	49.4	3.9	58.1	2.2	64.1	1.9	71.0	1.8	77.9	1.6	82.6	1.4
Female	56.1	3.7	62.2	2.2	69.3	2.1	73.8	1.9	81.1	1.7	83.5	1.6
By age group												
<1	8.7	10.5	28.0	6.3	27.0	5.2	46.2	6.5	52.0	6.5	64.1	5.5
1–4	64.3	4.5	68.4	2.2	76.4	1.9	78.4	1.7	87.4	1.4	89.3	1.3
5–9	53.2	5.0	67.3	3.0	72.2	2.7	74.4	2.4	82.0	2.1	83.6	2.0
10–14	40.5	4.7	43.2	3.5	48.0	3.5	63.5	3.5	64.7	3.2	73.7	2.9
Lymphomas												
Both sexes^a^	60.9	3.8	72.6	2.8	76.1	2.6	84.2	2.1	84.8	2.2	88.7	1.8
NHL^a^	54.4	6.1	65.4	4.4	75.0	3.8	74.4	3.8	81.7	3.2	84.5	2.6
												
*Relative survival*												
Leukaemias												
Both sexes^a^	52.8	2.8	60.8	1.6	66.6	1.4	72.4	1.3	79.2	1.2	83.0	1.1
By type^a^												
ALL	60.8	3.1	67.5	1.7	74.3	1.5	79.2	1.4	85.7	1.2	88.2	1.0
AML	20.7	4.8	29.5	4.4	35.8	4.4	40.5	3.8	49.0	3.7	60.8	3.7
By sex^a^												
Male	49.5	3.9	58.2	2.2	64.2	1.2	71.1	1.8	78.1	1.6	82.7	1.4
Female	56.2	3.7	62.3	2.2	69.4	2.1	73.9	1.9	81.2	1.7	83.6	1.6
By age group												
<1	8.8	10.5	28.2	6.3	27.2	5.2	46.6	6.6	52.4	6.6	64.6	5.5
1–4	64.4	4.5	68.5	2.2	76.5	1.9	78.5	1.7	87.6	1.4	89.4	1.3
5–9	53.3	5.0	67.4	3.0	72.3	2.7	74.5	2.4	82.1	2.1	83.6	2.0
10–14	40.6	4.7	43.2	3.5	48.1	3.5	63.6	3.5	64.8	3.2	73.9	2.9
Lymphomas												
Both sexes^a^	61.0	3.8	72.7	2.8	76.2	2.6	84.3	2.1	84.9	2.2	88.8	1.8
NHL^a^	54.4	6.1	65.5	4.5	75.1	3.8	74.5	3.8	81.8	3.2	84.6	2.6

Abbreviations: ALL=acute lymphoblastic leukaemia; AML=acute myeloblastic leukaemia; NHL=non-Hodgkin lymphoma; SEER=surveillance, epidemiology and end results.

a Adjusted to the age distribution of the leukemia/lymphoma of the patients from the Philippines.
